# Compartment Syndrome of the Hand: A Little Thought about Diagnosis

**DOI:** 10.1155/2016/2907067

**Published:** 2016-05-12

**Authors:** Eric F. Reichman

**Affiliations:** Emergency Department, University of Texas Health Science Center at Houston, Medical School, Houston, TX 77030, USA

## Abstract

Compartment syndrome of the forearm is a well described entity but there have been relatively few case reports in the emergency medicine literature of hand compartment syndromes (HCS). Prompt recognition and treatment of this potential limb threat are essential to minimize morbidity and mortality. Presented is a case of a documented hand compartment syndrome following a motor vehicle collision.

## 1. Introduction

In the setting of the emergency department, we are used to assessing common injuries and fractures. However, the potential complications of these injuries, while less frequent, must always be considered and ruled out. Although not as common as compartment syndromes of the forearm and leg, a compartment syndrome of the hand is not rare and can lead to adverse sequelae.

A compartment syndrome can develop in the hand from a variety of causes that decrease the size of the compartment, increase the volume of fluid in the compartment, or do both. The ability to diagnose a compartment syndrome is a critical skill for the emergency physician. Early identification of a compartment syndrome can enable the appropriate treatment and may facilitate functional recovery.

A thorough understanding of the intracompartmental anatomy of the hand is essential to recognize a compartment syndrome in the hand. The diagnosis is based upon clinical signs and assisted by intracompartmental pressure measurements. Direct pressure measurements offer the objective means of the diagnosis and can be performed using various techniques. It can be particularly challenging to detect a compartment syndrome, especially in the obtunded patient [1]. Effective treatment of a compartment syndrome requires early diagnosis and prompt surgical intervention to avoid disastrous complications. Delays in treatment are more likely to result in devastating functional loss to the patient.

A compartment syndrome can occur in almost any muscle group that is contained within a confined fascial space. Identifying a compartment syndrome in a timely fashion can be challenging. The sensitivity and specificity of manual palpation to identify a compartment syndrome are poor [2]. Thus, the use of manual palpation cannot be used to rule in or rule out a compartment syndrome. The hallmark symptom is persistent and progressive pain that is disproportionate to the underlying cause. The pain typically increases with passive motion. A catastrophic mistake is to attribute the etiology of the patient's pain solely to the underlying problem, such as the fracture or trauma [3, 4]. Other signs and symptoms associated with a compartment syndrome occur late in the course and include paresthesias of the involved nerve, paralysis of the involved muscle group, pallor of the skin, and diminished pulses. Waiting for the development of all the clinical signs and symptoms is an invitation for permanent and dangerous sequelae, including muscle necrosis and possible loss of a limb. Measurement of elevated tissue pressure within the muscle compartment is currently the most common objective means of diagnosing this syndrome. Compartment pressure measurement provides objective support for the diagnosis of a clinically suspected compartment syndrome.

## 2. Case Presentation

The patient is a 37-year-old Asian male who is right hand dominant. He initially presented to an outside emergency department (ED) after a motor vehicle collision. He was restrained in the front passenger seat when his vehicle rear-ended another vehicle. He extended his right upper extremity against the dashboard just before the impact. At the first ED, the patient was noted to have right wrist tenderness as well as lacerations over the dorsal surfaces of the 2nd and 3rd digits. The degree of swelling and presence of tenderness were not noted. Plain radiographs were obtained. They were read as notable for fractures of the distal radius, scaphoid, triquetral, and capitate bones. An anterior dislocation of the lunate bone was also present. Closed reduction of the lunate dislocation was attempted unsuccessfully a total of three times. The hand lacerations were then irrigated and sutured closed. The hand was placed in a splint in the position of function (intrinsic plus). He was then discharged on Cephalexin and acetaminophen with codeine and instructed to follow up at our ED for further evaluation later that day.

The patient was seen in our ED approximately 19 hours after discharge from the first hospital ED. The history was confirmed. The right hand and forearm were noted to be swollen, tense, and very tender. Light touch and two-point discrimination were diminished but present. Capillary refill was 2 seconds in all digits. The motor exam was remarkable for marked diminution of strength secondary to pain. Radiographs obtained confirmed the presence of the fractures noted at the first hospital. Compartment pressures were measured using a Stryker Intracompartmental Pressure Monitoring System in select areas of the forearm and hand. They were significantly elevated in all compartments tested (Table 1).

The orthopedic surgery service was consulted. The patient was taken to the operating room where he underwent release of the transverse metacarpal ligament, numerous fasciotomies of the dorsal interosseous compartments of the hand, and a fasciotomy of the forearm volar compartment. The compartment pressures were measured using a Stryker Intracompartmental Pressure Monitoring System. All the involved compartments became soft and compartment pressures normalized. The scaphoid, radius, and triquetral bones were pinned. This was followed by reduction of the lunate dislocation. Postoperatively the patient did well. He returned to the operating room one week after the original surgery for closure of the fasciotomy wounds and a split thickness skin graft. He attended outpatient occupational and physical therapy. He regained full sensory and fine motor control of his hand within three months of the injury.

## 3. Discussion

A compartment syndrome is defined as an elevation of the interstitial pressure in a closed fascial compartment resulting in microvascular compromise. Areas with noncompliant structures have a higher risk of becoming involved. These include the forearm and deep leg compartments. However, a compartment syndrome can develop in any area where there is muscle tissue surrounded by a fascial lining.

This case demonstrates some of classic findings described by Matsen et al. associated with compartment syndromes [5]. These include pain, paresthesias, pulselessness, paralysis, and pallor. Our patient presented with three of the five P's with pallor and pulselessness being absent. Complicating this case was the fact that the patient was outside of the medical system for 19 hours. It is not known when he first began to develop increased compartment pressures. Additionally, there is the question of whether the original injury, the failed reduction attempts, or the combination of both led to the development of the hand compartment syndrome.

The pathophysiologic insult of a compartment syndrome is decreased tissue perfusion within a closed osteofascial compartment [6]. As interstitial pressures increase, eventually it will exceed capillary perfusion pressure. The increased local venous pressure leads to a narrowed arteriovenous perfusion gradient resulting in a reduction of muscle perfusion below the level necessary for cellular viability. This leads to local hypoxia, nerve dysfunction, and muscle necrosis [7]. Results of canine model experiments show onset of tissue necrosis and arrest of nerve conduction after 8 hours of tissue pressures above 40 mmHg [8].

**Figure 1:**
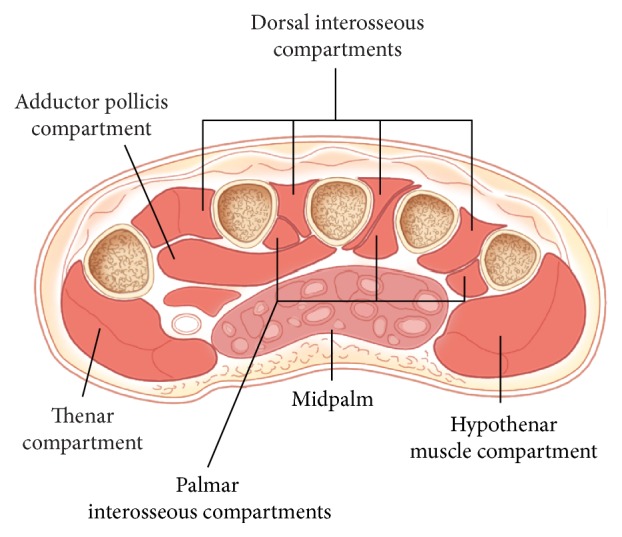
The eleven compartments of the hand [12].

The hand is made up of eleven separate compartments with some slight anatomic variations [5, 9–11]. There are four dorsal interossei compartments, three volar interossei compartments, a thenar compartment, a hypothenar compartment, an adductor compartment, and the midpalm compartment (Figure 1). The blood supply is provided by branches from the deep and superficial arches which are fed by the radial and ulnar arteries, respectively [11].

The causes of a hand compartment syndrome are varied (Table 2) and result from a wide spectrum of injury [5, 6, 10, 13, 14]. Crush injuries, fractures, snake bite, hemophilia, and burns are some of the more common causes. Intravenous or intra-arterial injections as well as local anesthetic blocks have all been implicated. Compression dressings, excessive exercise, and intrauterine umbilical cord strangulation are some of the lesser known but reported etiologies of hand compartment syndromes.

The diagnosis of a compartment syndrome is primarily a clinical one [5, 6, 13, 14]. In cases where the diagnosis is in question, intracompartmental pressures may be a useful supplemental tool. The most common presenting symptom is pain which is worse when the involved muscles are stretched passively [5]. The pain is usually severe and grows progressively worse. Swelling and palpable tenderness over a compartment are other early signs. Pulselessness and pallor often imply arterial involvement through either arterial compression or transection [15]. Paresthesias and paralysis typically are late signs and indicate some degree of nerve ischemia. In contrast to compartment syndromes at other sites, a hand compartment syndrome generally lacks the neurologic findings such as sensory deficits as no nerves are located within the hand compartments [16]. In cases where the diagnosis is in question, intracompartmental pressures may be a useful supplemental tool. Direct measurement of intracompartmental pressure may be particularly useful in the intoxicated, obtunded, uncooperative, or unreliable patient.

There is debate as to what compartment pressure measurement should mandate a fasciotomy. No absolute threshold pressure exists over which a fasciotomy is indicated. This is only of relevance in cases where the patient is obtunded and a clinical exam is not feasible or in patients where the exam is inconclusive. One can proceed with the fasciotomy on clinical grounds alone [15]. The prevailing thinking is that it is not so much the absolute compartment pressure that is important as it is the intracompartmental perfusion pressure [12].

The following guidelines for fasciotomy have been recommended [10, 12]. Operative fasciotomy is indicated if compartment pressures are within 30 mmHg of the diastolic pressure, within 45 mmHg of the mean arterial pressure, or greater than 30 mmHg. While awaiting transport to the operating room the hand should not be elevated. This will only serve to decrease tissue perfusion without decreasing the compartment pressures and potentially worsening the ischemia. Once in the operating room, the involved muscle compartments are released by incising the fascial coverings.

In the setting of a hand compartment syndrome it is often necessary to make multiple incisions to facilitate access to all areas [5, 14]. The most common approach involves a carpal tunnel release, two longitudinal dorsal incisions, and a thenar eminence incision. Usually the wounds are left open and skin closure is delayed 3–5 days.

## 4. Conclusion

A compartment syndrome is a well-documented phenomenon. The clinical presentation is variable and changes over time. It is a difficult clinical diagnosis that is critical for the emergency physician to make in a timely fashion. While the most common sites are the lower leg and forearm, a compartment syndrome can occur in any muscle compartment of the body.

Compartment syndromes of the hand are rare but occasionally the emergency physician will be afforded with the opportunity to make this diagnosis and avert significant morbidity for the patient. A high clinical suspicion is important to recognize an evolving compartment syndrome of the hand. A marked increase in pain, swelling, and pain with passive stretch are the hallmarks of the diagnosis. Sensory deficits and perfusion abnormalities may be absent. Clinical suspicion and the physical examination are the most important criteria for treatment, supplemented by compartment pressure measurements to aid in the diagnosis.

Determining the pressure within a compartment is a fundamental and essential tool to aid in this diagnosis. Many methods exist for the measurement of compartment pressures. Any concern for a compartment syndrome should be followed up with an emergent orthopedic surgeon or general surgeon consultation, as continuous observation and repeated measurements are often indicated. Pressures over 30 mmHg or within 30 mmHg of the diastolic blood pressure warrant an emergent evaluation for a possible fasciotomy and limb salvage. Any hand crush injury that swells and is tense should alert the physician to the possibility of a compartment syndrome, as not all compartment syndromes present with the classic signs and symptoms [17].

Failure to act quickly, make the diagnosis, and perform operative intervention followed by aggressive postoperative rehabilitation often results in devastating consequences including necrosis, amputation, Finochietto's ischemic retraction, and nerve paralysis.

## Figures and Tables

**Table 1:** The patient's measured compartment pressures. Only representatives of the compartments were measured.

Compartment	Pressure (mm Hg)
Volar forearm	40
Dorsal forearm	35
Thenar space	60
2nd interosseous space	45
4th interosseous space	45
Hypothenar space	55
Dorsal hand	52

**Table 2:** The varied etiologies of a compartment syndrome [1, 3, 5–7].

Etiology	Examples
Burns	Electrical, thermal
Coagulopathies	Bleeding disorders, Coumadin, hemophilia, heparin
Iatrogenic	Arterial line placement, closure of fascial defects, embolectomy, fracture reduction, intravenous line infiltration, orthopedic surgery, prolonged operating room positioning, prolonged tourniquet use, tight casts and splints, tight dressings
Infection	Gas gangrene, necrotizing fasciitis
Miscellaneous	Cardiac catheterization, ergotamines, intra-arterial drug injections, immobility, intravenous infiltration, nephrotic syndrome, reperfusion injury, tetany, venous occlusion
Overuse syndromes	Exercise, weight lifting
Trauma	Bleeding, contusions, crush injuries, fractures, gunshot wounds, high pressure injection injuries, seizures, snake bite
